# Dr. Hamilton, on the Effects of Ipecacuanha

**Published:** 1810-04

**Authors:** R. Hamilton

**Affiliations:** Ipswich


### *To the Editors of the Medical and Physical Journal.*

Gentlemen,

COLLECTIONS of insulated facts may in time form the basis of general rules, or prove the key for the discovery of general laws in Nature. Periodical works like yours, therefore, where every fact may be speedily recorded, become their proper repositories. The number of individuals who are willing to undergo the labour of composing long dissertations, or entering into deep reflections, on professional subjects, is comparatively few. Scarcely an individual, however, engaged in practical medicine, but will have it in his power on various occasions, with little trouble, to add some facts to the accumulating store. He has only carefully to observe, and note down what is unusual, or new to him, as it recurs, to be transmitted to your Journal, or some other of the same sort, as early as convenient.

A fact in your Journal for June last, and another in the one last published, on the pernicious Effects of Ipecacuanha to certain individuals, induced me to send you a similar communication.

Mr. Spencer, indeed, on whose apprentice this drug exerted its anomalous effects, invites the profession to communicate such similar instances as have come to their knowledge. His communication appears to be the first, and the subject has lain dormant till Mr. B. in your last Journal, has added a second, as occurring in his own person; and you, Sir, mention a third in the person of your own father.

To these three cases in the male subject, I shall now add two in the female, who seem to have been affected in so similar a manner by the subtle effluvia of ipecacuanha, that to enumerate their symptoms would be only to repeat what what has already been given, respecting those effects above mentioned.

The first of these cases is that of a lady, now about 50, the wife of a surgeon, and mother of a numerous family. The general state of health has always been good, her disposition lively and active, and by no means possessing any thing of that valetudinarian irritability which marks striking peculiarity of constitution. She has been much in the habit, when the hurry of business required it, of assisting her husband in dispensing medicines. This gave rise to her first discovery of the effects of ipecacuanha on her habit.

I had an opportunity of remarking this fact about eighteen months ago, being on a professional visit at her house, while her husband laboured under a severe fever.

She was about to dispense one of my prescriptions, in which some ipecacuanha had been ordered, and the moment she saw what the composition was, she ran from the shop to a distant part of the house, refusing to dispense it. This excited my curiosity to find the cause. On following her, she explained it, and with some degree of anxiety looked round, lest some of the doors between her and the shop should have been left open while the prescription was about to be dispensed.

As my stay was protracted some days, I had occasion to see these fears repeatedly excited. One forenoon in particular, while she was in her kitchen, a considerable distance from the shop, (two passage doors being open between herself and it) while she could neither see nor know before hand, that ipecacuanha, which was the case, was weighing, she called out with vehemence to have the doors closed, on account of the sensations she was then beginning to feel.

The second instance came to my knowledge only the day before yesterday. The lady who is the subject of it, calling on me on her mother's account, who was indisposed, and being shewn into my room, took up your last Journal, which lay on my table, to amuse herself till my appearance, as I was at that time engaged on some family affairs, which detained me a little. On my entering the room, she told me she had been reading my book, and the part which she accidentally opened, was Mr. B's communication. She added with a smile, this is far from so uncommon a case as this gentleman seems to think, for I myself am affected by it in the same manner; and then went into a considerable detail of the symptoms it excited in her.

The catarrhal affection and sneezing she described as particularly distressing. The copious flow was so acrid, as to excoriate, in a few hours, the parts over which it fell. Her upper lip and the alæ of the nostrils were swelled. But what created in her the most alarm was its effects on her eyes. They became proportionably swelled, and stiff, and sight was diminished. The eye-lids tumefied, so that the eyes were sunk almost out of sight, which seemed to be the chief cause of the diminution of vision. The discharge from her eyes was nearly as great as that from her nose, and little less acrid. Thus was she tormented for that day, but found herself relieved next morning by a night's rest. The remaining effects, the itchings excepted, gradually disappeared, and in a day or two ceased altogether. She felt likewise a tightness in the chest, which greatly impeded respiration; but as she was occasionally affected with a temporary inconvenience of this kind, her sensations were only an augmentation of that symptom, and could not therefore be altogether attributed to the ipecacuanha, though this evidently brought on a severe recurrence. The lady is about 33, unmarried, and of a somewhat delicate and irritable habit, tending to valetudinarian. She discovered its effects on her in the following way. Her late father, a dissenting minister, got into the habit, for several years before his death, from benevolent principles, (whether erroneous or otherwise, is not the business of this place) of prescribing for the poor, and on the Buchan and Reece principle, kept a medicine chest in his house. Gratis practice has generally a numerous attendance, and his, if not altogether, was nearly gratis. This lady was in the habit of assisting him to dispense; and in this manner discovered the effects of this drug on herself. At first she attributed the symptoms to cold, not having any suspicion respecting the powder, but her reiterated feelings on every occasion of dispensing it, convinced her at last, that they must have arisen from its effluvia.

I am therefore of opinion with this lady; and from these five cases now before you, think that we only want observations and consequent communications to convince us that the pernicious effects of ipecacuanha, from its effluvia taken into the trachea and lungs, are far from unusual.

This lady, who possesses an excellent understanding, and a philosophical turn of thinking, put the thing in some measure to the test, by comparing it with the effluvia of of another irritating substance well known to induce sneezing and a flow of mucous when applied to the Snidereian membrane of those unaccustomed to its stimulus, as she was. Being soon after in company, where there were persons in the habit of taking snuff, she placed herself, as if by accident, between two of them, of her own sex. The good ladies had been long initiated in this filthy and often pernicious, though fashionable custom, and as they took it plentifully, dispersed its finer particles around, so that she inhaled an atmosphere thickly impregnated with it. No catarrhal effects were excited, and scarcely any sneezing, though it was otherwise disagreeable to her organs of smell.

Her conclusion was, that the effects she felt from the ipecacuanha, were in consequence of a peculiar quality inherent in it beyond a simple stimulus.

In my turn therefore, I now solicit your readers to communicate such instances of it as may have fallen under their observation.

I am, &c.

R. HAMILTON.

*Ipswich, March* 12, 1810.

